# Supplementary material for: Identifying SARS-CoV-2 antiviral compounds by screening for small molecule inhibitors of nsp15 endoribonuclease
Source: Biochem J. 2021 Jul 2;478(13):2465–79. doi: 10.1042/BCJ20210199 (PMC8286823; doi:10.1042/BCJ20210199)
Supplement: Supplementary Figures S1-S4 and Tables S1-S4 [file BCJ-478-2465-s1.pdf]

Supplementary Material

Supplementary Figure S1. Size exclusion chromatography of purified SARS-CoV-2 nsp15.

A. Each of the indicated protein preparations were loaded onto a 120 ml Superdex200 column, which has a void volume of ~40 ml. Approximate protein amounts as measured by UV_{280} are plotted versus accumulated elution volume. Note the presence of the later-eluting peak of the partially cleaved 14His-SUMO-nsp15 preparation, consistent with the untagged nsp15 existing as a smaller complex or monomer. **B**. Fractions from the size-exclusion chromatography of A for the partially digested 14His-SUMO-nsp15 were run on SDS-PAGE and stained with coomassie.

Supplementary Figure S2. SARS-CoV-2 endoribonuclease nsp15 inhibitor screen design and results.

A. Titration of the 6 nt U substrate (0 – 1000 nM) in the presence of 75 nM nsp15 enzyme. Fluorescence quantified in a Spark Multimode microplate reader (Tecan) at RT every min for 60 min. **B**. Schematic of the distribution of the controls (substrate-only, enzyme-only and control reactions) and of the compound reactions (reactions + compounds) in the plates of the screen. **C**. Z' factor for each plate at both compound concentrations. **D**. Ordered Z-scores for sample wells at both concentrations.

Supplementary Figure S3. Fluorescence quenching test for screen hit compounds.

Graphs show the fluorescence of a 35 nt Cy5-oligonucleotide (Supplementary Table S2) in the presence of a titration of the top hit compounds from the screen (except compound 6). In red, compounds that quench Cy5 fluorescence.

Supplementary Figure S4. Validation of screen hit compounds in plate reader.

Nuclease reactions in the presence of 75 nM nsp15, 500 nM 6 nt U substrate and 10 µM of the 12 non-quenching selected screen hits (Supplementary Figure S3, except 6). Up top graph shows three control reactions (2 +DMSO, 1 +water). The rest of the graphs show control reactions (in black) and the reactions in the presence of the different compounds (in blue -no inhibition-, and in red -inhibition-). Water control reactions are shown for compounds 4 and 5, that were dissolved in water. Residual activity for each compound was calculated from this experiment and shown in Figure 4A.

Supplementary Figure S1

A

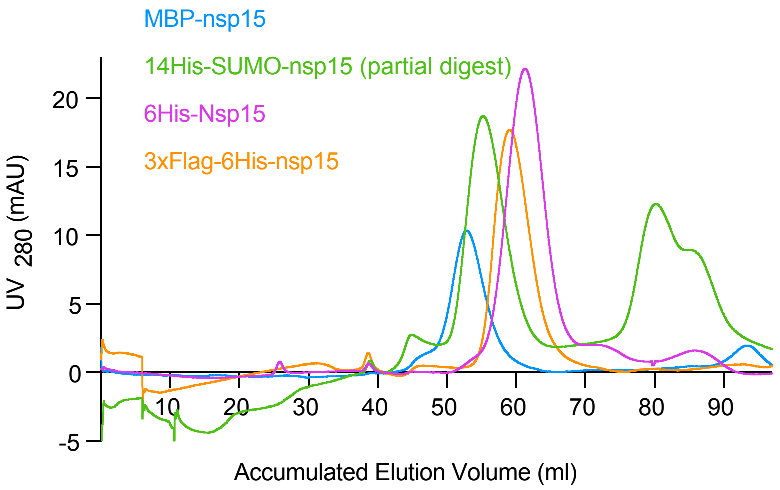

B

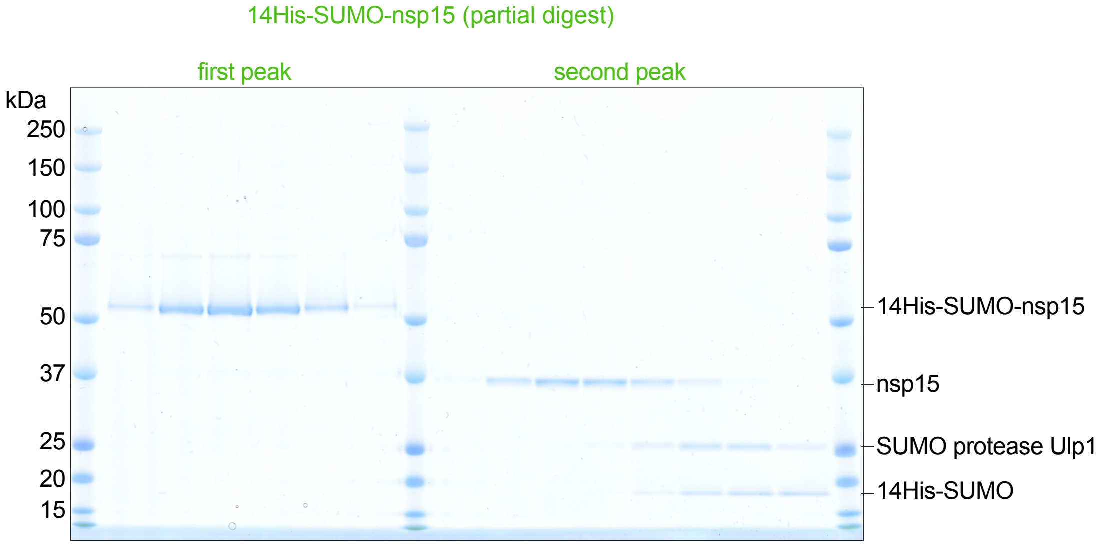

Supplementary Figure S2

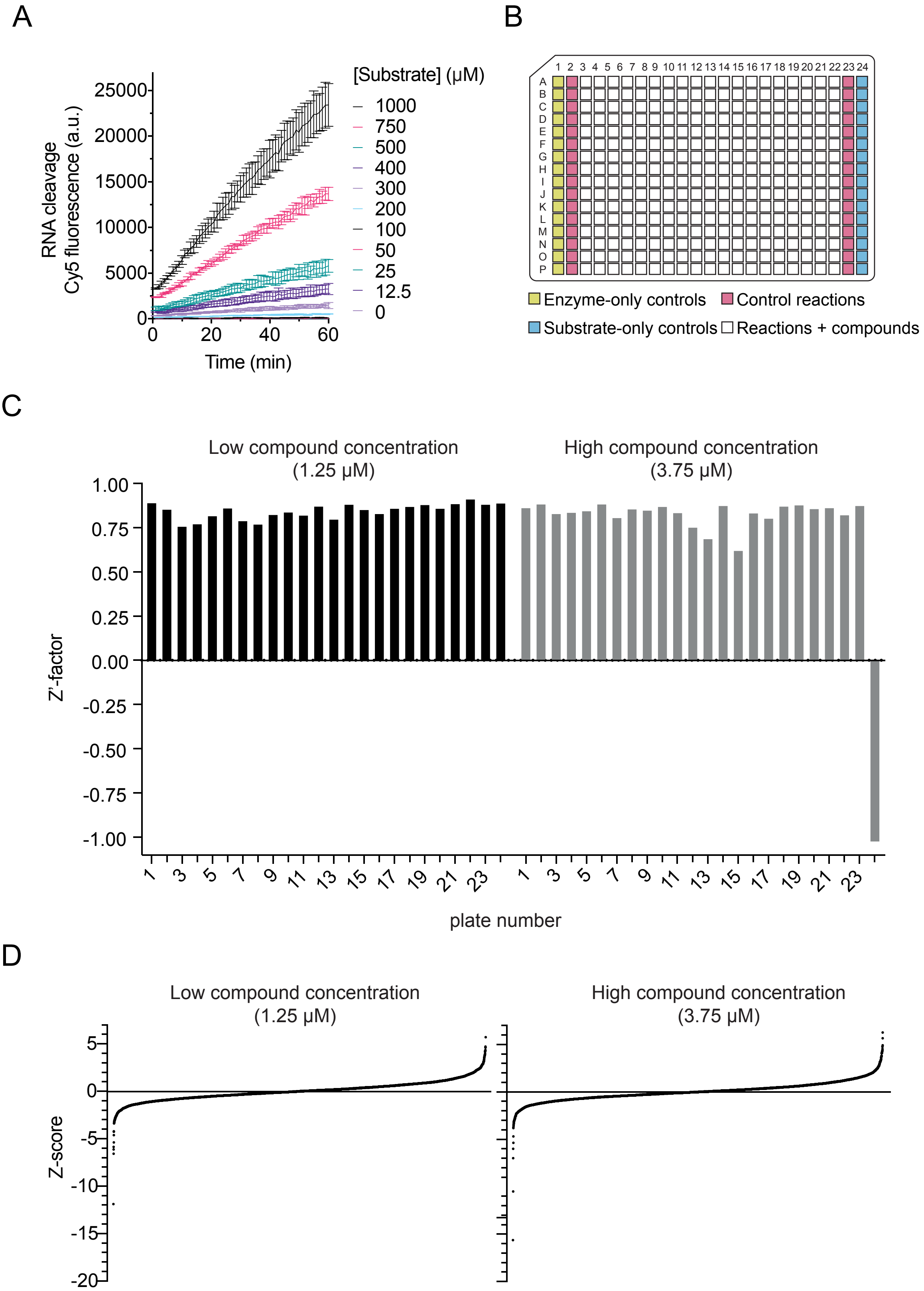

Supplementary Figure S3

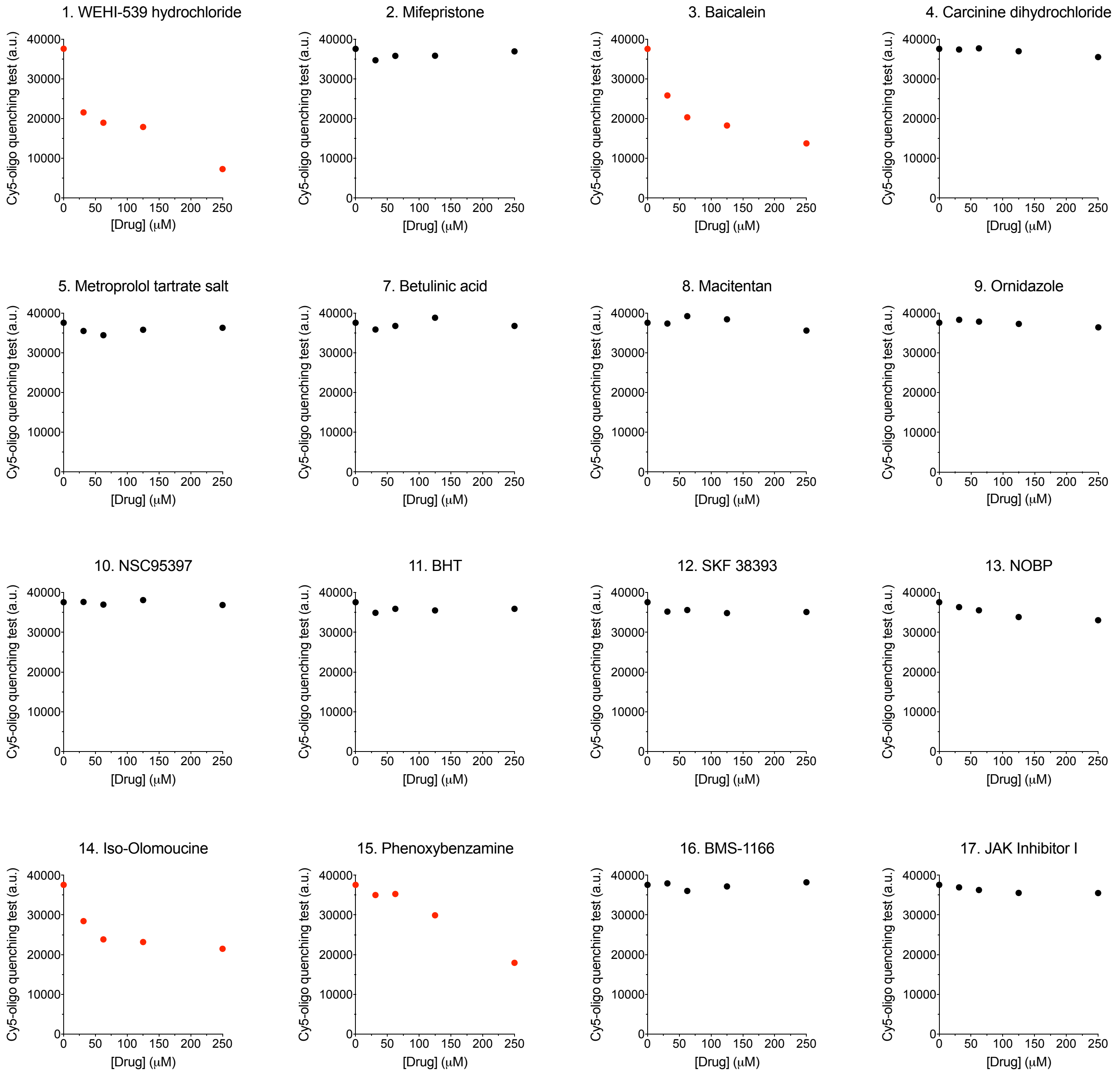

Supplementary Figure S4

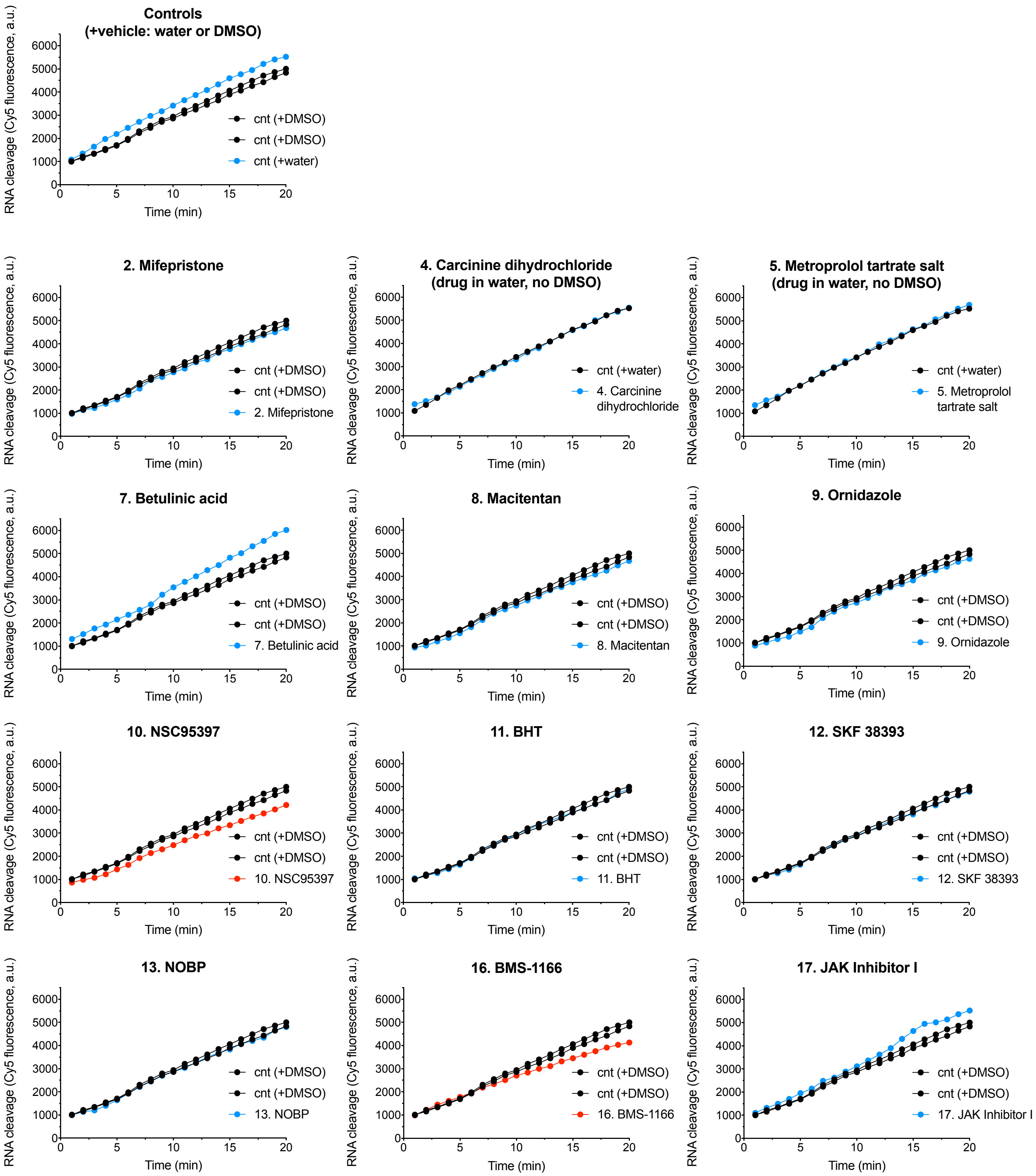

Supplementary Table S1. Constructs for protein expression			
**Expression**	**Construct ID**	**Available from**	**Cloning**
Constructs for expression in *E. coli*			
SARS-CoV-2 MBP-nsp15	DU67734	MRC PPU	Codon optimised SARS-CoV-2 nsp15 (Genbank MN908947.3) for bacterial expression, cloned into BamHI-NotI MCS of pMEXCb vector (https://mrcppu-covid.bio/cdna-clones/134146)
SARS-CoV-2 14His-Sumo-nsp15	DU70489	MRC PPU	PCR with oligos 9142/9143 from DU67734, Gibson assembly into K27 vector
SARS-CoV-2 6His-nsp15	DU70490	MRC PPU	PCR with oligos 9156/9157 from DU67734, Digest PCR fragment with NcoI and XhoI ligated into pET28C
SARS-CoV-2 6His-nsp15 (H234A H249A)	DU70491	MRC PPU	PCR with oligos 9150/9151 from pRF061
Constructs for expression in baculovirus-infected Sf9 insect cells			
SARS-CoV-2 3xFlag-6His-nsp5CS-nsp15	169166	Addgene	PCR with oligos FW701, FW702, FW758, FW759 from codon optimised nsp15 cloned into pBIG1a plasmid.

Supplementary Table S2. Oligonucleotides (cloning and assay substrates)			
**Name**	**Oligo sequence**	**supplier**	**purpose**
9142	gaacagattggtggcAGCCTGGAAAACGTG GCGTTTAAC	sigma	subcloning into K27 vector (14His-SUMO)
9143	gtgcggccgcttattaCTGCAGTTTCGGATAA AAGGTTTC	sigma	subcloning into K27 vector (14His-SUMO)
9150	TTAGCCATAGCCAGCTGGGCGGCCTGg cTCTGCTGATTGGCCTG	sigma	H234H249A mutagenesis
9151	GGCTATGGCTAAAATCGCCATACACAA TAgcTTCAAACGCATAGCC	sigma	H234H249A mutagenesis
9156	ATCAGTccATGggcCATCACCATCATCAC CATagcAGCCTGGAAAACGTGGCGTTTA ACG	sigma	subcloning into pET28C vector (6His)
9157	acgatgCTCGAGttaCTGCAGTTTCGGATA AAAGGTTTCCACATG	sigma	subcloning into pET28C vector (6His)
FW701	CCACCATCGGGCGCGGATCCAGCCAC CATGGACTACAAGGACCACGACGGCG ATTACAAGGATCATGACATCGACTACA	sigma	N-3FHcs-PEP_for
FW702	GCAGCACAGCAGAGTGATGGTGGTGA TGGTGGGAACCCTTGTCATCGTCGTCT TTGTAGTCGATGTCATGATCCTT	sigma	N-3FHcs-PEP_rev
FW758	TCCTCTAGTACTTCTCGACAAGCTTTTA CTGCAGCTTGGGGTAGAAAGTC	sigma	nsp15_rev
FW759	ATCACTCTGCTGTGCTGCAGTCCCTGG AAAACGTGGCCTTCA	sigma	3FH-nsp15_for
16 U substrate	5’-Cy3-CAGACAAACU/AAGAAC-3’	eurofins	16 nt "U" RNA substrate for gel-based nuclease assay
16 C substrate	5’-Cy3-CAGACAAACC/AAGAAC-3’	eurofins	16 nt "C" RNA substrate for gel-based nuclease assay
6 U substrate	5’-Cy5-CAU/AAC-BHQ650-3’	eurofins	6 nt "U" RNA substrate for Plate reader-based nuclease assay
6 C substrate	5’-Cy5-CAC/AAC-BHQ650-3’	eurofins	6 nt "C" RNA substrate for Plate reader-based nuclease assay
Quench test substrate	5'-Cy5-TCAGCTAGATATCATCGTCGTATCAAGT GCGCTAC	eurofins	35 nt Cy5-only oligo for Plate reader-based quenching assay

Supplementary Table S3. Top screen hit compounds					
**Compound number**	**Chemical Name**	**CAS No**	**Aggregation Index (LogP)**	**Lowest Residual activity**	**Lowest Z-score**
1	WEHI-539 hydrochloride	2070018-33-4	6.80	0.56	-6.98
2	Mifepristone	84371-65-3	5.60	0.85	-2.33
3	Baicalein	491-67-8	2.7 (100%*)	0.80	-3.06
4	Carcinine dihydrochloride	57022-38-5	-1.50	0.88	-2.36
5	Metroprolol tartrate salt	56392-17-7	2.00	0.76	-4.62
6	GNF-PF-3777**	77603-42-0	2.50	1.21	3.95
7	Betulinic acid	472-15-1	7.00	0.03	-15.63
8	Macitentan	441798-33-0	4.00	0.69	-5.86
9	Ornidazole	16773-42-5	0.10	0.66	-5.37
10	NSC 95397	93718-83-3	1.10	0.62	-5.99
11	BHT	128-37-0	5.4 (83%*)	0.77	-4.27
12	SKF 38393 hydrobromide	20012-10-6	1.9 (75%*)	0.65	-6.59
13	NOBP	130506-22-8	1.90	0.68	-6.13
14	Iso-Olomoucine	101622-50-8	6.2 (80%*)	0.35	-10.49
15	Phenoxybenzamin e	63-92-3	4.40	0.37	-11.88
16	BMS-1166	1818314-88-3	4.20	0.81	-2.99
17	JAK Inhibitor I	457081-03-7	0.50	0.72	-5.38

* % similarity to known aggregator **chosen for high score as putative nsp15 activator

Supplementary Table S4. Screen results of known and predicted nsp15 inhibitors					
**Chemical Name**	**Residual activity [High]**	**Z-score [High]**	**Residual activity [Low]**	**Z-score [Low]**	**Reference**
Congo red	0.93	-0.97	0.94	-1.10	Ortiz-Alcantara et al 2010
***In silico* predicted nsp15 inhibitors**					
Novobiocin	0.98	-0.19	0.95	-0.97	Kumar et al 2020
Sarsasapogenin	1.00	0.19	0.96	-0.79	
Piperine	0.99	0.05	0.97	-0.64	
Gingerol	1.03	0.67	0.98	-0.46	
Curcumin	0.95	-0.69	1.00	-0.04	
Ursonic acid	1.01	0.30	1.03	0.56	
Silymarin	1.01	0.37	1.00	0.05	
Rosmarinic acid	1.06	1.19	1.01	0.08	
Ergotamine	0.99	0.06	1.00	0.03	Barage et al 2020
Dihydroergotamine mesylate	0.95	-0.71	0.98	-0.33	
Idarubicin	0.97	-0.30	0.97	-0.61	Chandra et al 2020
